# Correction: A bis-macrocyclic peptide ligand mimicking the β-sandwich active site of multicopper oxidases

**DOI:** 10.1039/d6dt90087a

**Published:** 2026-06-11

**Authors:** Viet Thuc Dang, Alexander Drena, Joshua Telser, Lane Wofford, Andy I. Nguyen

**Affiliations:** a Department of Chemistry, University of Illinois Chicago Chicago IL 60607 USA andyn@uic.edu; b Department of Chemistry, Northwestern University Evanston IL 60208 USA; c College of Science, Health, and Pharmacy, Roosevelt University Chicago IL 60605 USA

## Abstract

Correction for ‘A bis-macrocyclic peptide ligand mimicking the β-sandwich active site of multicopper oxidases’ by Viet Thuc Dang *et al.*, *Dalton Trans.*, 2026, **55**, 5877–5881, https://doi.org/10.1039/D6DT00494F.

In the published version, the affiliations of Andy I. Nguyen were incorrectly listed as “a,c”. The correct affiliation of Andy I. Nguyen should only be “a”, which is University of Illinois Chicago. In addition, the word “macrocyclic” in the title was spelled incorrectly as “macrocylic”. The corrected title and list of authors and affiliations for this paper are as shown herein.

The Royal Society of Chemistry apologises for these errors and any consequent inconvenience to authors and readers.

